# Stable Isotope and Element Profiling for Determining the Agroclimatic Origin of Cow Milk within a Tropical Country

**DOI:** 10.3390/foods11030275

**Published:** 2022-01-20

**Authors:** Maheshika Kalpage, Champa Dissanayake, Saranga Diyabalanage, Rohana Chandrajith, Russell Frew, Ruchika Fernando

**Affiliations:** 1 Sri Lanka Atomic Energy Board, Orugodawatta, Wellampitiya 00900, Sri Lanka; maheshika@aeb.gov.lk (M.K.); champa@aeb.gov.lk (C.D.); 2 Instrument Center, Faculty of Applied Sciences, University of Sri Jayewardenepura, Nugegoda 10250, Sri Lanka; saranga@sjp.ac.lk; 3 Ecosphere Resilience Research Center, Faculty of Applied Sciences, University of Sri Jayewardenepura, Nugegoda 10250, Sri Lanka; 4 Department of Geology, Faculty of Science, University of Peradeniya, Peradeniya 20400, Sri Lanka; rohanac@pdn.ac.lk; 5 Department of Chemistry, University of Otago, Dunedin 9016, New Zealand; russell.frew@otago.ac.nz; 6 Department of Veterinary Public Health and Pharmacology, Faculty of Veterinary Medicine and Animal Science, University of Peradeniya, Peradeniya 20400, Sri Lanka

**Keywords:** origin authentication, cow milk, stable isotopes, elemental profiling, agroclimatic zones, Sri Lanka

## Abstract

Information on the geographic origin of milk is important in determining quality attributes and for economic gain through building brand value associated with origin. Stable isotope signatures and trace element concentrations have been increasingly used in authentication of milk, though information on the power of such technology in verifying agroclimatic origin in small continents with diverse climatic, environmental conditions, and animal management practice is scarce. Therefore, the main objective of this study was to investigate the possibility of using a stable isotope composition of C, N, O, and H and element fingerprints to determine the agroclimatic origin of milk produced in different agroclimatic zones of Sri Lanka. Stable isotopes ratios of C, N, H, and O, and elemental fingerprints of milk samples were determined by IRMS and ICP-MS, respectively. Significant variations were observed in stable isotope ratios, especially δ^18^O and the mean content of Li, Al, Cr, Mn, and Sr in the bulk milk samples obtained from different agroclimatic zones. A linear discriminant analysis differentiated cow milk produced from four agroclimatic zones based on stable isotope ratios, and the inclusion of elemental ratios enhanced the discriminating ability.

## 1. Introduction

Authentication of geographical origin is a rapidly emerging topic in food safety, quality, traceability, and consumer protection. Tools to validate the geographic origin of food including milk are extremely valuable, as the origin is often associated with quality attributes, for instance, free from disease and pollution. Recent scares, such as melamine adulteration of milk powder [1], have focused wider public attention on the importance of knowing the origin of milk.

Various analytical techniques, including liquid and gas chromatography, isotope ratio and elemental analyses, spectroscopy, DNA, and sensor techniques, have been widely used to authenticate the geographical origin of various foodstuffs [2]. Among these techniques, trace element concentrations and stable isotope signatures have been increasingly applied in authentication studies, since their chemical patterns have a direct causal relationship to geographical factors, such as the environment in which the products were grown and/or processed. Stable isotope signatures mainly depend on climatic or geographical conditions, while the elemental composition is primarily affected by geological and pedological characteristics of the soil [2,3,4]. These techniques have been widely adapted to determine geographic authenticity of various foodstuff, including honey, wines, olive oil, spirits, fruit juices, essential oils, and milk [5,6,7,8,9,10]. Isotope ratio analysis has been successfully applied in the verification of geographic origin of milk, with different dietary regimes and production systems [11,12]. Isotope ratios of oxygen and hydrogen are commonly used for regional origin assessment, and carbon and nitrogen stable isotope ratios are used for determining the feeding practices of animals [13]. Several studies have combined the stable isotope ratios with multi-element analysis in determining the origins of milk and dairy products. It was found that the stable isotope ratios in milk from Australia, New Zealand, and Austria have different isotopic signatures and a multivariate model with stable isotope ratios, elements, and fatty acids has successfully differentiated the origins of milk with a good predictive ability [14]. Further, a study conducted with δ^13^C and δ^15^N values of the proteins extracted from milk samples originating from four continents, including Australia (with New Zealand), Europe (Germany and France), North America (USA), and Asia (China) has provided evidence about their potential as “fingerprints” of the geographical origin of milk [15]. Fatty acid δ^2^H and δ^13^C have been provided as good biomarkers in milk powder to trace back milk powders to their regional origin in a study conducted in New Zealand [16]. Further, another feasibility study conducted in New Zealand has shown that the δ^13^C and δ^15^N isotopes of milk casein reflect the inherent geo-climatic variation across dairy farms sampled in the south and north islands [17]. Several studies conducted with milk samples collected from various provinces in China have successfully shown the ability of stable isotopes to verify the geographic origin [18,19].

Most of these studies have been carried out by major dairy exporters, such as Europe, New Zealand, Australia, and Argentina with mostly temperate breeds and more uniform and large-scale dairy operations compared to the South Asian region. In South Asia, the dairy industry is mostly comprised of small-scale farmers (with few to several animals either of tropical and/or temperate breeds) with varying management practices from free-grazing to intensive farming. So far limited work has been carried out on investigating the potential of stable isotope ratios and multi-element analysis in determining the origins of milk in the South Asian region.

Sri Lanka is a small island (65,610 km^2^) located south of India with a varied climate and topography. The cattle management practices in Sri Lanka vary across the country according to the climate, geographic location, crops grown and cropping pattern, availability of grazing land, and genetic make-up of the animals [20,21,22,23] (Table 1), and most of the dairy operations are of small scale. Factors, such as climate, altitude, latitude, vegetation, type of breed, and feeding and management practices may influence the isotope and elemental composition in milk. Therefore, the main objective of this study was to investigate whether a chemometric differentiation model based on the stable isotope composition and element fingerprints of cow milk can be successfully used to verify the agroclimatic origins of milk produced under the main four agroclimatic zones of Sri Lanka involved in dairy production. These widely accepted agroclimatic zones have been defined based on rainfall pattern, altitude, cropping system, and agroecology, hence there are differences in animal husbandry practices and breeds utilized among these agroclimatic zones (Table 1). Such information can be used as a foundation for investigations on the verification of the geographic origin of milk precisely to a small agroclimatic area.

## 2. Materials and Methods

### 2.1. Sample Collection and Preparation

**Figure 1:**
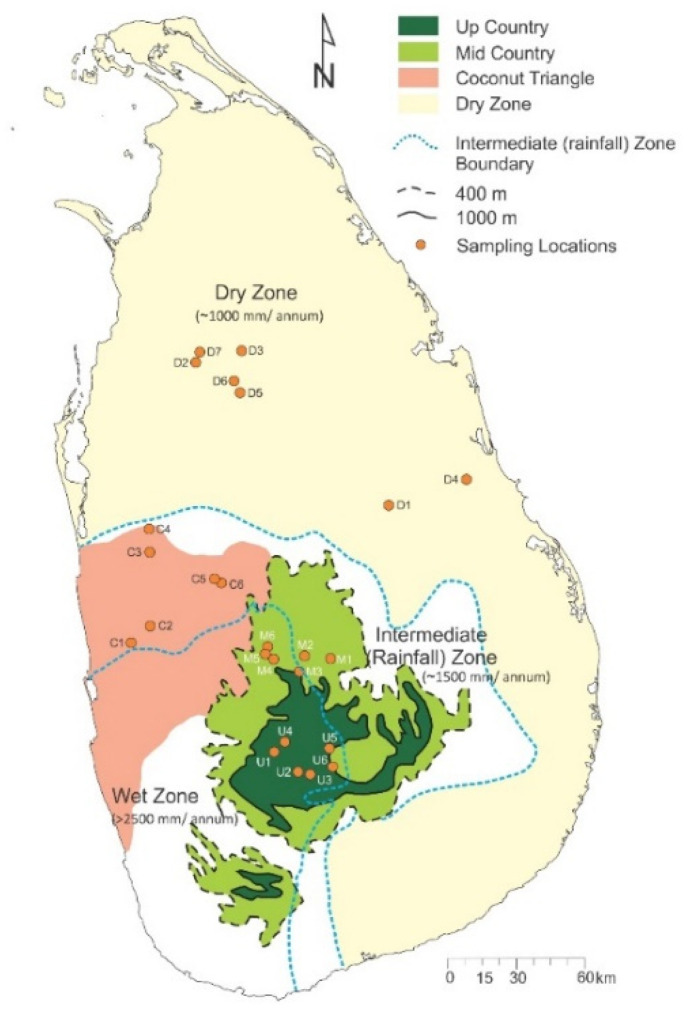
Milk sampling locations and agroclimatic zones of Sri Lanka.

Milk samples were collected from four different agroclimatic zones in Sri Lanka, including dry zone, coconut triangle, mid country, and up country (Figure 1). Six large to medium-scale dairy farms were selected from each of the agroclimatic zones for sampling except the dry zone. Seven farms/locations were selected in the dry zone as a contingency plan since one farm indicated that they were planning to convert their dairy farm to a buffalo farm. Two liquid milk samples of 250 mL were collected on three consecutive days from a selected farm for trace element and stable isotope analysis. The same farms were sampled during wet and dry periods. Samples were collected from the bulk collection tank immediately after milking into pre-cleaned (acid washed) high-density polyethylene bottles. Samples were transported in cooling containers with ice and then stored in a −20 °C freezer until analyses.

Milk samples were prepared based on the method proposed by Feng et al. [24] and validated by Luna et al. [25]. An aliquot of 1.5 mL of milk was centrifuged at 20,000× *g* for 30 min at 4 °C. With the aid of a pipette, without disturbing the fat layer the skim milk fraction in the bottom of the tube was transferred into a micro-centrifuge tube. The initial tube with milk fat was saved to analyze stable isotope ratios of milk fat. Then 6.0 M HCl (about 60 µL) was added to the skim milk sample to facilitate precipitation of milk casein at its isoelectric point (pH 4.6). Next, the micro-centrifuge tube was vortexed for 5 min and allowed to settle for 10 min at room temperature to facilitate casein precipitation. Finally, the tube was centrifuged at 20,000× *g* for 30 min at 4 °C to precipitate milk casein. The supernatant containing the whey fraction was transferred to another tube to separate the milk whey fraction. All these fractions (fat, casein, and milk whey) of the samples were stored at −20 °C until lyophilization.

### 2.2. Stable Isotope Analysis

For δ^13^C and δ^15^N analysis, whole milk, milk fat, milk casein, and whey were measured as 1.0 ± 0.05 mg, 0.5 ± 0.05 mg, 1.0 ± 0.05 mg, and 2.0 ± 0.05 mg into tin capsules, respectively, and encapsulated. The encapsulated samples were placed in a desiccator until the isotope ratio mass spectrometer (IRMS) analysis was performed. Bulk δ^13^C and δ^15^N were measured by combustion to CO_2_ and N_2_, respectively, using Euro-EA Elemental Analyzer (EuroVector). The isotope ratio of the gases was measured on a Europa 20–20 isotope ratio mass spectrometer (Sercon Ltd., Cheshire, UK) operating in continuous flow mode. The isotopic compositions of the sample gases were normalized and reported against the international scales for carbon and nitrogen, VPDB, and ambient air. Normalization was made by three-point calibration with international standards, USGS 40 (L-glutamic acid, δ^13^C = 26.24‰ and δ^15^N = −4.52‰), USGS 41 (L-glutamic acid, δ^13^C = 37.76‰ and δ^15^N = 47.56‰), and EDTA-OAS laboratory standard (Elemental Microanalysis Ltd., Cornwall, UK, δ^13^C = −38.93‰ and δ^15^N = −0.73‰). Time-based drift correction was calculated by placing laboratory standards (IAEA 153 milk powder) with EDTA-OAS after every 12 samples. Precision was assessed by placing sequential duplicates of the samples after every 10th sample.

For δ^18^O and δ^2^H analysis, 0.6 ± 0.05 mg of bulk milk samples were weighed into silver capsules which were closed loosely to allow the exchange of water vapor for correction of the effect of exchangeable hydrogen. Capsule bottoms were slightly flattened to keep them standing upright. Samples were left for six days in ambient conditions to achieve equilibrium of exchangeable hydrogen with the lab atmospheric water. Three replicates were prepared for each sample.

Samples were placed into the autosampler carousel. Spaces were left for polymer standard materials (IAEA-CH7, Kilwell, Nylex, Shah Alam, Malaysia). The carousel was placed in a desiccator and evacuated to remove air. Samples were vacuum dried on a hot plate set to 40 °C for four days. IAEA Milk Powder 153 (δ^2^H = −40.78 ± 5.2‰, δ^18^O = 16.81 ± 0.59‰), USGS-53 (Lake Shala Distilled Water, δ^2^H = +40.2 ± 0.4‰, δ^18^O = +5.47 ± 0.03‰), USGS 47 (Lake Louise Drinking Water, δ^2^H = −150.2 ± 0.5‰, δ^18^O = −19.80 ± 0.02‰), CH7 (δ^2^H = −99.2‰), and IAEA601 (Benzoic Acid, δ^18^O = 23.14‰) were used as the reference samples. CH7 and IAEA601 were repeated as control samples at each 15th position throughout the batch for drift correction by linear regression against time. Control samples and USGS-53 were prepared on the same day in a separate tray. Hydrogen and oxygen isotope ratios of the samples were determined by pyrolysis to H_2_ and CO gases in a Thermal Conversion Elemental Analyzer (TC/EA, Thermo Scientific, Bremen, Germany), followed by isotope analysis of the gas using Thermo Delta-V IRMS in continuous flow mode. Raw results were corrected to the international VSMOW (Vienna Standard Mean Ocean Water) isotope scale using two-point calibration. The measurement uncertainty was calculated by analyzing six to eight replicates of the quality control standard interspersed regular intervals (after every 12 samples) in every batch of samples analyzed throughout the sample analysis period. The measurement uncertainty reported herein is the highest uncertainty value obtained for a single batch of samples throughout the entire sample analysis. The uncertainty values were ±0.18‰ for δ^13^C, ±0.25‰ for δ^15^N, ±0.32‰ for δ^18^O, and ±1.46‰ for δ^2^H.

The isotopic difference between the sample and an international standard reported in delta notation (δ) units in parts per thousand (‰) is determined by the formula [26]:

(1) δ (Ei/j)=Rpi/j−RRefi/jRRefi/j

where ^i^E denotes the higher and ^j^E the lower atomic mass number of element E. The subscript P denotes the samples used to determine the respective values. R indicates the isotope number ratios and Ref indicates the reference material; Vienna Pee Dee Belemnite (V-PDB) for carbon (δ^13^C), atmospheric N_2_ (Air-N_2_) for nitrogen (δ^15^N), and VSMOW for δ^18^O and δ^2^H.

### 2.3. Trace Element Analysis

An aliquot of 1 mL of each milk sample was digested with 5 mL of supra-pure HNO_3_ (>69%, Fluka, Switzerland) and 1 mL of H_2_O_2_ (35%, Sigma-Aldrich, Darmstadt, Germany) at 200 °C for 10 min in a MARS-6 Microwave digester (CEM Mathews, Charlotte, NC, USA) equipped with an EasyPrep Plus high-pressure vessel system. The resulting solutions were then diluted to 25 mL with deionized water (<0.055 µScm^−1^) and stored in polypropylene bottles at 4 °C until analysis.

Digested samples were filtered through 0.45 µm cellulose acetate membrane filters, and concentrations of trace elements were measured using Thermo ICapQ high-resolution ICP-MS (Thermo Fisher, Bremen, Germany). Before the analysis, the instrument was optimized with a solution containing Ba, Bi, Ce, Co, In, Li, and U to cover the entire mass range and obtain high sensitivity. A series of multi-element standards prepared by diluting 10 mg L^−1^ AccuStandard^®^ (New Haven, CT, USA) stock solution was used to calibrate the instrument. Internal calibration was carried out using 100 µg L^−1^ of ^103^Rh and ^185^Re standards. All measurements were conducted at least in triplicate, and analytical results were expressed as mean values. Analytical quality controlling was assessed using Certified Reference Material BCR-063R (skim milk powder from the Institute for Reference Materials and Measurements, European Commission). The given reference values and test values showed that the recoveries of available elements ranged from 85–115%, and relative standard deviations by sextuplicate analyses of elements were less than 10%. Further, synthetic external standards, duplicates, and reagent blanks were analyzed at every 10th sample and measurement uncertainties were always less than 2%. Deionized water blanks were also analyzed recurrently to check for sample carry-over.

### 2.4. Statistical Analysis

Results of isotope ratios and elemental concentrations were statistically evaluated using R-Studio version 3.3.0 and SAS (version 9.1; SAS Institute Inc., Cary, NC, USA). Data were preliminarily explored by preparing box plots and scatter diagrams. Descriptive statistics were taken for each data set. One-way analysis of variance (ANOVA) was used to assess stable isotope data to evaluate whether the variables used differ between the agroclimatic zones, the sampling period’s effect, and their interaction. The means were separated using Duncan’s multiple range tests at a confidence level of 95%. Principle component analysis (PCA) was performed to check if the combined isotopic parameters could be used to distinguish different agroclimatic zones. The linear discriminant analysis (LDA) test was performed with a *p*-value of ≤0.05 to discriminate samples according to agro-climatic zones based on the elemental composition and isotope ratios as well as isotopic data alone. The predictive ability of the LDA model was evaluated by leave-one-out cross-validation.

## 3. Results and Discussion

### 3.1. Stable Isotopic Composition in the Milk and Its Constituents 

**Figure 2:**
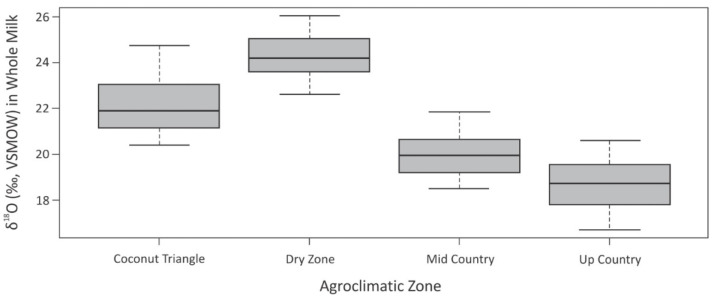
Box-and-whisker plot of δ^18^O in whole milk samples collected from agroclimatic zones of Sri Lanka.

Stable isotope ratios of 142 milk samples collected from four different agroclimatic zones of Sri Lanka varied from −15.5 to −28.0‰ for δ^13^C, 11.6 to 3.3‰ for δ^15^N, −48 to −155‰ for δ^2^H, and 26.0 to 16.7‰ for δ^18^O. The mean δ^18^O of whole milk samples showed a significant difference among the four agroclimatic zones (Table 2). Milk from the dry zone region showed relatively higher δ^18^O values than the other zones, while up country milk showed the lowest values (Figure 2). 

**Figure 3:**
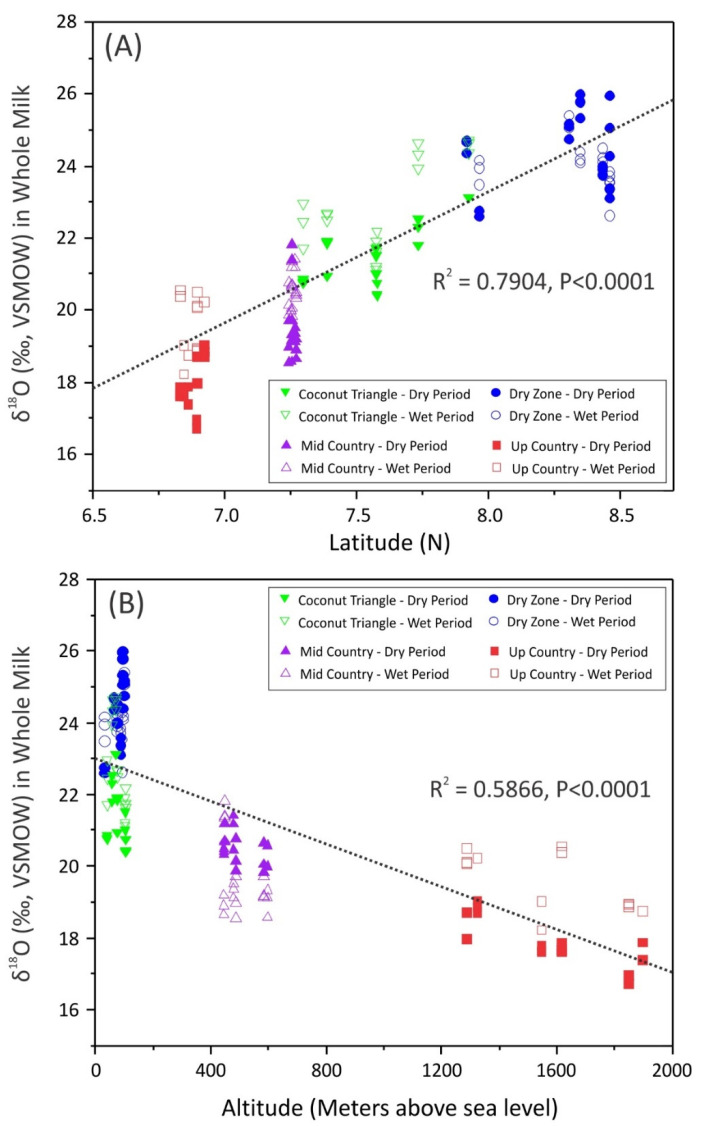
Correlation between δ^18^O values of whole milk samples collected and farms at different altitudes and latitudes. (**A**) Variation of δ^18^O in whole milk with latitude, (**B**) variation of δ^18^O in whole milk with altitude.

The present study showed a significant correlation of δ^18^O values with latitude, a moderate correlation with altitude (Figure 3), and a lower correlation with distance to the coast. It has been revealed that the isotopic enrichment of exchangeable oxygen and hydrogen in plants and animals is usually shifted in relation to the local precipitation, as the enrichment of all sources of absorbed water is altered by plant or animal metabolism. Temperature is an important factor governing the enrichment of δ^18^O and δ^2^H in precipitation [27]. The milk samples collected from Sri Lanka showed higher δ^18^O values at lower elevation and lower values at high elevation. This observation could have resulted from the influence of climatic factors, such as temperature and rainfall, as relative humidity affects the fractionation of oxygen isotopes. Each agroclimatic zone receives rainfall from different rainfall regimes by the two main monsoon rains and two convectional (inter-monsoon) rains known to have characteristic isotopic signatures [28]. The isotopic composition of monsoon rains in Sri Lanka is affected by terrestrial and oceanic moisture sources, depression, and cyclonic conditions of the Bay of Bengal, Arabian Sea, and the country’s topography [29].

Higher evapotranspiration rates in the dry zone may also lead to enriched δ^18^O values in plants and animals. In Sri Lanka, the temperature decreases at a steady rate of about 6.5 °C for each 1000-m rise. Higher temperatures are experienced generally in the dry zone areas of northern, northcentral, and eastern regions of the island that range between 33.3–34.7 °C [30]. The dry zone receives a mean annual rainfall of less than 1000 mm with a distinct dry season, while the intermediate zone receives a mean annual rainfall of about 1750 mm. The wet zone receives over 2500 mm of annual rainfall [30]. 

In the present study, the δ^15^N values of whole milk, milk casein, and whey revealed a moderate potential for discriminating cattle farming zones. However, this study found that δ^15^N as well as δ^13^C values of dry zone samples were significantly different from other zones (Table 2). The highest δ^15^N values were reported in the dry zone samples, and the lowest δ^15^N values were reported in the up country. The whole milk δ^15^N values were between their casein and whey δ^15^N values for all the agroclimatic zones except the upcountry. In up country, the whole milk δ^15^N values were higher than their respective casein and whey δ^15^N values. Further, up country recorded the lowest temperature range (18–23 °C) and the other zones showed a higher temperature range (27–34 °C) during sampling. According to literature, ambient temperature could influence casein δ^15^N values [17]. The isotopic fractionation of nitrogen in dairy cows was estimated to be high in metabolic origin and affected by temperature as the metabolic rates of animals are indirectly affected by environmental temperatures. In up country, animals are subjected to minimal heat stress compared to the other zones. 

Complete milk protein is composed of 20% whey protein and 80% casein. δ^15^N values of milk whey were more depleted than that of milk casein and whole milk in all four agroclimatic regions. A similar observation has been reported by Kornexl et al. [31]. This finding may be attributed to the different amino acid compositions and relative abundance of amino acids originating from body tissues or diet [31,32]. Further, the δ^15^N value in agricultural products is influenced by the differences in soil conditions, amount of rainfall, soil age, nitrogen turnover rate in soils, and other complex parameters [17,31]. Regional differences in the δ^15^N value of milk may reflect the differences in feeding regimes depending on the regional dairy farm [11]. There is a trend for increasing foliar δ^15^N with decreasing rainfall [33].

**Figure 4:**
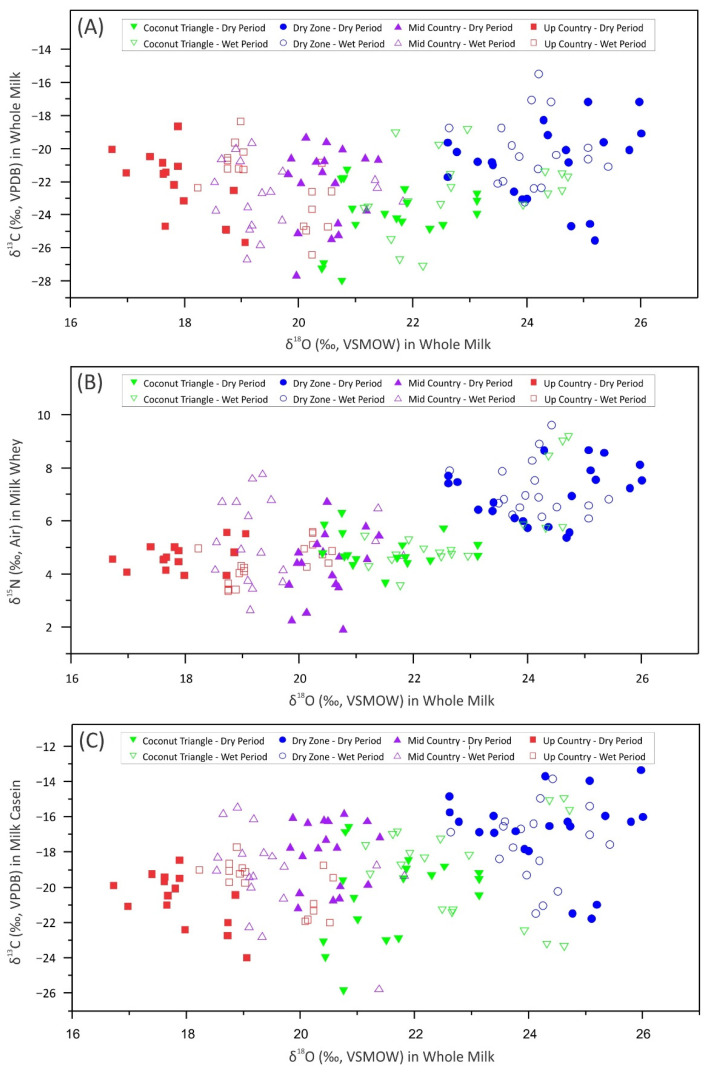
Scatter diagrams of distribution of stable isotopes values. (**A**) δ^18^O ‰ in whole milk vs. δ^13^C ‰ in whole milk, (**B**) δ^18^O ‰ in whole milk vs. δ^15^N ‰ in milk whey, (**C**) δ^18^O ‰ in whole milk vs. δ^13^C ‰ in milk casein.

The difference in δ^13^C reflects the relative amounts of C3 and C4 plants used by the regional feeding regimes. According to the Calvin cycles in photosynthetic CO_2_ fixation, C3 plants show δ^13^C values between −24 and −32‰. Plants following the C4-dicarboxylic acid pathway show δ^13^C values between −10 and −19‰ [34]. Except in the dry zone, all other cattle management zones used particularly the grasses of the genera *Brachiaria* and *Panicum* to feed cattle, which fall into the C4 plant category. *Gliricidia sepium*, which is in the C3 category, was fed to cattle of all the zones except the up country. In up country, Ryegrass (*Lolium multiflorum*) was given to the animals which is also a C3 plant. However, a considerable amount of concentrate feed, such as rice bran, rice polish, coconut poonac were given as cattle feed in most of the farms. Cattle reared in dry zone eat various types of plants, while freely grazing on communal grazing lands. Therefore, the δ^13^C values showed a value in between C3 and C4 plants in cattle feed for most of the samples. However, in this study, the δ^2^H value displayed weak potential to differentiate milk samples of different agroclimatic zones. The variations in δ^18^O, δ^13^C, and δ^15^N values for each of the agroclimatic zone investigated are presented in Figure 4. This representation helps visualize the potential of stable isotope values as a ‘screening’ parameter to differentiate milk produced in different agroclimatic zones. Samples from the dry zone could be visualized separately from the mid country and up country.

### 3.2. Influence of Sampling Period on Stable Isotope Values of Milk

Although δ^13^C, δ^15^N, δ^18^O, and δ^2^H in whole milk and milk components were significantly influenced by agro-climatic zone, these variables were also moderately influenced by the sampling period (Table 3) and less influenced by interactions of the sampling period and the region. Mean δ^18^O values in whole milk displayed a significant difference in wet and dry sampling periods for each agroclimatic zone except for the dry zone (*p* < 0.001). The only variable that displayed a significant difference in wet and dry periods for the dry zone was δ^15^N of milk casein (*p* < 0.001). Significant variation between sampling periods in the δ^15^N values of milk casein and whole milk samples collected from coconut triangle and up country was observed. Mean δ^15^N of whole milk and milk fractions and δ^18^O showed relatively enriched values for the wet period compared with the dry period.

Forage production and availability in Sri Lanka mainly depend on the rainfall. The availability of lush pastures is high during rainy season and forage is scanty during dry season. Due to the scarcity of forage during the dry season, cattle are often fed with different feeding materials, such as silage and concentrate feeds. The difference of δ^15^N during wet and dry seasons may have resulted from variations in ambient temperature and feed material [17,31].

The topographical features and regional scale monsoons wind regimes dominate the climate dynamics in each agroclimatic zone. Monsoonal, convectional, and depressional rain contribute to the seasonal rainfall in Sri Lanka. The topographical features of the various agroclimatic zones mean they receive rain in different months of the year. The two main monsoonal rains and two inter-monsoon rains have characteristic isotopic signatures [28]. The up country agroclimatic zone falls in the most affected area of the southwest monsoon. North-east monsoon and second inter-monsoon rains are the significant rainy seasons in mid country. The dry zone and coconut triangle receive abundant rainfall from the northeast monsoon [30]. The source of precipitation, temperature, isotopic depletion of moisture in clouds moving inland, elevation effect, and cooling of the vapor masses as they rise over the landscape may change the isotopic signatures of δ^18^O and δ^2^H in different months of the year in agroclimatic zones.

### 3.3. Determination of Geographical Origin by Statistical Analysis

The combined isotope parameters obtained from milk samples were evaluated to distinguish agroclimatic zones using multivariate statistics. The first two principal components explained 69% of the total variance (Table 4).

**Figure 5:**
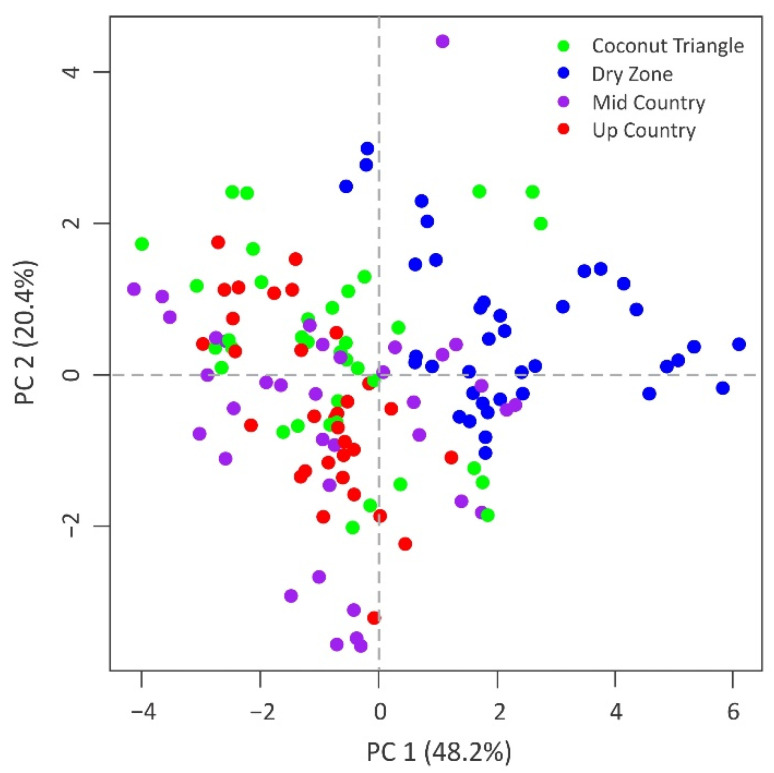
Scatter plot of the PC-1 and PC-2 based on the principal components analyses of stable isotopes ratios.

However, clear clustering was not observed in the score plot of PC1 and PC2 (Figure 5). As the PCA could not clearly group the samples based on the origin, LDA was employed with backward stepwise refinement of stable isotope ratios to provide a mathematical model to classify samples simultaneously according to their agroclimatic origin. The selected variables in the model were whole milk δ^15^N, milk casein δ^15^N, milk whey δ^15^N, whole milk δ^13^C, milk fat δ^13^C, milk casein δ^13^C, whole milk δ^2^H, and whole milk δ^18^O. The efficacy of the model in discriminating milk samples originated in coconut triangle, dry zone, mid country, and up country from the rest agroclimatic regions were 83%, 92%, 89%, and 97%, respectively. After cross-validation, the above percentages were 75%, 92%, 81%, and 91%, respectively. Table 5 summarized original and cross-validated classification by discriminant analysis for the samples collected from four different agroclimatic zones. The discriminant analysis also showed a degree of overlap. As illustrated in Figure 1, farms located closer to the border areas of these agroclimatic zones share similar climatic conditions. 

Samples collected during the dry period were evaluated for elemental composition and stable isotope ratios. Previous studies have shown that there is a correlation between the elemental profile of animal products and geographic origin. Further, the elemental profiles of milk have shown to be a useful tool for verification of geographic origin [35,36,37]. The elements present in the natural environment go through a complex metabolic process inside the feed (eg., grass) and animal, and the variability of trace elements in milk may be affected by both intrinsic and extrinsic factors related to feed and the animal. A significant variation in climate, soil, and cattle management practices can be observed between the agroclimatic zones of Sri Lanka, though geographic distances between these zones are small (Figure 1). Al, Cr, Mn, Fe, Co, Ni, Cd, and Pb may be related to intrinsic factors of the animal, such as breed, stage of lactation, parity, and health status together with feeding and dietary adaptations based on the availability of feeding material. Li, Cu, Zn, As, Se, Sr, Ba, and Pb could be attributed to dietary supplements and feed additives used to feed animals [38,39,40,41,42]. Se, Cu, and Zn are present in mineral mixtures fed to intensively manage high yielding animals [40]. However, there are very limited data available on correlations between elemental content of milk and geographic origin for the South Asian region. The value of such information is vital to study the applicability of elemental content analysis for geographic origin verification for the region, as cattle management practices vary widely in the region even within the same agro-climatic zone.

**Figure 6:**
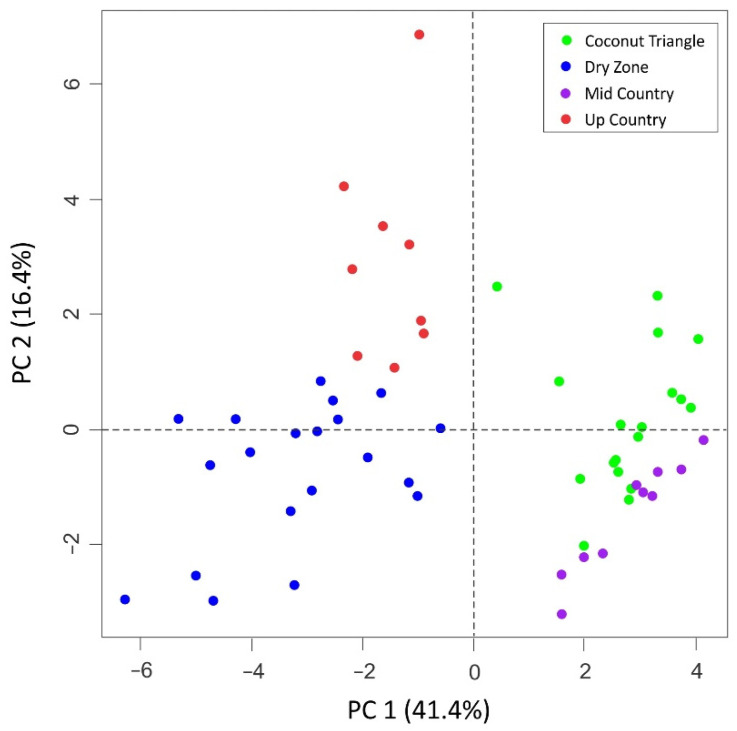
Scatter plot of the PC-1 and PC-2 based on the principal component analysis of stable isotopes and trace element data.

**Figure 7:**
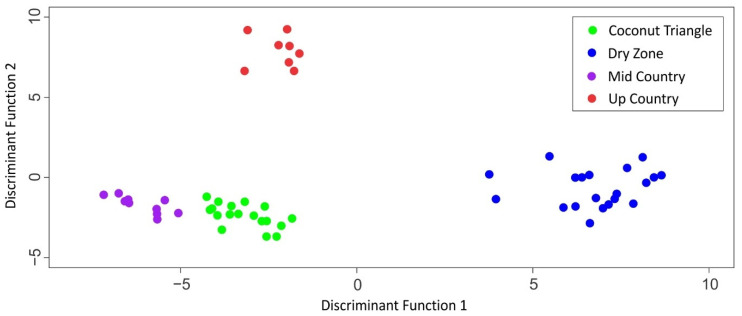
LDA of milk samples with both selected trace elements and stable isotope ratios to discriminate four agroclimatic zones.

Geologically, underlying over 90% of Sri Lanka is Precambrian high-grade metamorphic rocks which can be subdivided into three main litho-tectonic units, namely Highland Complex, Vijayan Complex, and Wanni Complex. The remaining 10% is underlain by Miocene sedimentary rocks. Rocks underlain in the Highland Complex are mainly granulite facies suites, including charnockites, garnet-biotite gneisses, quartzite, marbles, and calc-silicate gneisses. The Vijayan region consists of gneisses and granitoid varieties, while the Wanni Complex is dominated by supracrustal rocks and meta-igneous suits, such as granodioritic gneisses. All the sampling locations of up country and mid country, and D1 and D4 locations of the dry zone belong to the Highland Complex of the metamorphic terrain while other sampling locations belong to Wanni Complex. The major and trace element content in the natural environment is mainly determined by their content in rocks, which significantly varies within a very short distance in a metamorphic terrain like Sri Lanka [43]. However, it is difficult to directly correlate the local geology with the resulted elemental contents in milk, as there are other factors influencing the elemental content in milk such as management practices. Li, Al, Cr, Mn, and Sr contents in samples collected in the dry period indicated significant differences in their mean contents among four agroclimatic zones (Table 6). Fe, Ni, Cu, Zn, As, Se, Ba, Pb, and Bi displayed moderate potential in differentiating agroclimatic zones, and Co and Cd composition were similar among the different farming zones. Sr and Mn concentrations together with isotope ratios have been used as markers for geographical traceability of raw milk in previous studies [36,37]. The first two principal components explained 58% of the total variance and clear clustering was observed in the score plot of PC1 and PC2 (Figure 6). The LDA carried out using both trace element and stable isotope data with backward stepwise variable selection (i.e., milk casein δ^15^N, whole milk δ^15^N, milk casein δ^13^C, whole milk δ^18^O, whole milk δ ^2^H, Li, Al, Cr, Mn, Fe, Co, Ba, and Sr) showed excellent discrimination in samples from the dry zone, coconut triangle, mid country, and up country (100%) even after cross-validation (Figure 7).

One limitation of the study is that the authors could not select farms in each of the agro-climatic zones with a good geographic distribution, due to unavailability of suitable farms throughout the zones. This uneven distribution of farms resulted from suitable agricultural land, water sources and other infrastructure availability, and environmental considerations. In this study, only large to medium scale farms were selected. Even though some of these farms were very close to the agroclimatic boundaries, they could be clearly categorized into respective agroclimatic zones in the LDA carried out with trace elements and stable isotopes data. This could have been possible due to the differences in elemental contents in the environment. However, we could not confirm whether the LDA model can differentiate milk coming from locations that were not sampled, especially in the eastern and southern regions of the dry zone where suitable farms are not available. 

## 4. Conclusions

This study has demonstrated the ability of stable carbon, nitrogen, oxygen, and hydrogen isotope ratios, and multi-element analysis of cow milk to verify the agroclimatic origin of milk in a small tropical island with a diverse climate, environmental conditions, and cattle management practices. This study provides preliminary information on the applicability of stable isotope and elemental content analysis in verifying agroclimatic origin of milk, even within a small geographic area with diverse conditions. The isotope signatures significantly varied in accordance with the agroclimatic origin of the milk samples and were moderately influenced by the sampling period, detailed data can be found in the Supplementary Materials. The δ^18^O was the most correlated stable isotope with the agroclimatic zone and the LDA analysis of stable isotopes (whole milk δ^15^N, milk casein δ^15^N, milk whey δ^15^N, whole milk δ^13^C, milk fat δ^13^C, milk casein δ^13^C, whole milk δ^2^H, and whole milk δ^18^O) correctly classified more than 75% of the milk samples to their respective agroclimatic zones after cross-validation. The inclusion of selected elemental concentrations with stable isotope data (milk casein δ^15^N, whole milk δ^15^N, milk casein δ^13^C, whole milk δ^18^O, whole milk δ^2^H, Li, Al, Cr, Mn, Fe, Co, Ba, and Sr) of the milk samples enhanced the discriminating power of the LDA model up to 100%. A combination of selected elemental concentrations and stable isotopic compositions analyzed by a chemometric model is a promising tool to verify agroclimatic origin of cow milk besides high variations in climatic conditions and cattle management practices. Further, the data generated in the present study are expected to be used as baseline data to develop a model for non-targeted screening for potential chemical contaminants, such as dicyandiamide (DCD) and adulterants.

## Figures and Tables

**Table 1:** Milk production zones in Sri Lanka (after [23]).

Zone Features	Dry Zone	Coconut Triangle	Mid Country	Up Country & Estate
**Animal types**	Indigenous cattle, Zebu cattle, and crosses	Crosses of exotic breeds, Zebu types, crosses of indigenous animals	Pure exotic animals and crosses, and Zebu crosses	Pure exotic animals and crosses
**Husbandry**	Free gazing, or nomadic-type Large herds or sedentary small/medium-sized herds	Medium-sized herds, limited grazing tethered under coconut palms and/or stall feeding	Mostly small herds, some tethering, stall feeding	Small to large herds, stall feeding
**Elevation (m)**	>0–450	0–450	450–1200	>1200
**Rainfall (mm)**	1000–1750	1500–2500	>2000	>2000
**Temperature (°C)**	21–38	21–38	10–32	10–32

**Table 2:** Variation of mean, minimum, and maximum values of δ^13^C, δ^15^N, δ^18^O, and δ^2^H in milk and its components in each agroclimatic region. Data represent the mean ± standard deviation. Values in each row with different superscript letters differ for *p* < 0.001.

Variable		Coconut Triangle	Dry Zone	Mid Country	Up Country
Whole milk δ^15^N	Mean	6.1 ± 1.4 ^B^	8.1 ± 1.3 ^A^	6.0 ± 1.4 ^B^	6.6 ± 1.7 ^B^
	Min	4.3	6.4	3.3	4
	Max	10.8	11.5	8.6	11.6
Milk casein δ^15^N	Mean	6.9 ± 1.5 ^B^	8.9 ± 1.2 ^A^	7.1 ± 4.8^B^	5.3 ± 1.1 ^C^
	Min	3.9	6.9	3.4	3.0
	Max	11.1	12.5	33.2	7.0
Milk whey δ^15^N	Mean	5.2 ± 1.3 ^B^	7.1 ± 1.0 ^A^	4.8 ± 1.4 ^BC^	4.5 ± 0.6 ^C^
	Min	3.6	5.4	2.0	3.4
	Max	9.2	9.6	7.7	5.6
Whole milk δ^13^C	Mean	−23.3 ± 2.2 ^AC^	−20.6 ± 2.2 ^A^	−22.6 ± 2.2 ^BC^	−22.1 ± 2.1 ^B^
	Min	−28.0	−25.5	−27.7	−26.4
	Max	−18.9	−15.5	−19.4	−18.3
Milk fat δ^13^C	Mean	−26.0 ± 2.3 ^B^	−22.8 ± 1.9 ^A^	−25.0 ± 2.3 ^B^	−25.3 ±2.0 ^B^
	Min	−30.3	−26.1	−29.6	−29.0
	Max	−21.2	−19.2	−22.0	−21.3
Milk casein δ^13^C	Mean	−19.6 ± 2.7 ^BC^	−17.1 ± 2.2 ^A^	−18.7 ± 2.3 ^B^	−20.2 ± 1.4 ^C^
	Min	−25.8	−21.8	−25.8	−24.0
	Max	−14.9	−13.3	−15.5	−17.8
Milk whey δ^13^C	Mean	−21.1 ± 2.2 ^B^	−18.9 ± 2.5 ^A^	−21.2 ± 2.1 ^B^	−20.9 ± 1.6 ^B^
	Min	−25.2	−24.5	−25.8	−23.5
	Max	−16.9	−14.3	−18.1	−18.0
Whole milk d^2^H	Mean	−101 ± 19 ^AB^	−93 ± 15 ^A^	−108 ± 21 ^B^	−93 ± 12 ^A^
	Min	−136	−129	−155	−124
	Max	−48	−63	−74	−72
Whole milk δ^18^O	Mean	22.2 ± 1.3 ^B^	24.2 ± 0.9 ^A^	20.0 ± 0.9 ^C^	18.7 ± 1.1 ^D^
	Min	20.4	22.6	18.5	16.7
	Max	24.7	26.0	21.8	20.6

**Table 3:** Variation of mean δ^13^C, δ^15^N, δ^18^O, and δ^2^H in milk and milk components in each agroclimatic region for samples collected at dry period and wet period (values of the wet and dry season in each agroclimatic zone with different superscript letters differ for *p* < 0.001).

Variable		Coconut Triangle		Dry Zone		Mid Country		Up Country	
		Dry Period	Wet Period	Dry Period	Wet Period	Dry Period	Wet Period	Dry Period	Wet Period
Whole milk δ^15^N	mean	5.5 ^b^	6.8 ^a^	7.9 ^a^	8.4 ^a^	5.8 ^a^	6.2 ^a^	5.9 ^b^	7.2 ^a^
	sd	±0.6	±1.7	±0.9	±1.6	±1.5	±1.2	±0.5	±2.1
Milk casein δ^15^N	mean	6.3 ^b^	7.4 ^a^	8.3 ^b^	9.7 ^a^	7.1 ^a^	7.1 ^a^	4.5 ^b^	6.3 ^a^
	sd	±1.1	±1.7	±0.9	±1.2	±1.6	±6.7	±0.4	±0.9
Milk whey δ^15^N	mean	4.9 ^a^	5.6 ^a^	7.0 ^a^	7.2 ^a^	4.3 ^b^	5.3 ^a^	4.4 ^a^	4.6 ^a^
	sd	±0.6	±1.6	±1.0	±1.0	±1.3	±1.5	±0.5	±0.7
Whole milk δ^13^C	mean	−24.0 ^a^	−22.6 ^a^	−20.9 ^a^	−20.1 ^a^	−22.8 ^a^	−22.3 ^a^	−22.2 ^a^	−22.1 ^a^
	sd	±1.8	±2.3	±2.3	±2.1	±2.4	±2.0	±2.0	±2.2
Milk fat δ^13^C	mean	−26.7 ^a^	−25.4 ^a^	−23.3 ^a^	−22.2 ^a^	−25.3 ^a^	−24.8 ^a^	−25.3 ^a^	−25.3 ^a^
	sd	±1.9	±2.5	±1.9	±1.7	±2.3	±2.4	±1.9	±2.1
Milk casein δ^13^C	mean	−20.4 ^a^	−18.8 ^a^	−17.5 ^a^	−16.7 ^a^	−19.3 ^a^	−18.1 ^a^	−20.7 ^a^	−19.9 ^a^
	sd	±2.5	±2.7	±2.3	±2.1	±1.9	±2.5	±1.5	±1.3
Milk whey δ^13^C	mean	−21.4 ^a^	−20.7 ^a^	−18.9 ^a^	−18.8 ^a^	−21.6 ^a^	−20.9 ^a^	−21.0 ^a^	−20.8 ^a^
	sd	±1.8	±2.5	±2.6	±2.5	±2.0	±2.2	±1.7	±1.6
Whole milk δ^2^H	mean	−111 ^b^	−92 ^a^	−93 ^a^	−93 ^a^	−110 ^a^	−106 ^a^	−94 ^a^	−91 ^a^
	sd	±14	±19	±15	±16	±17	±25	±9	±14
Whole milk δ^18^O	mean	21.6 ^b^	22.8 ^a^	24.1 ^a^	24.3 ^a^	19.5 ^b^	20.5 ^a^	17.9 ^b^	19.5 ^a^
	sd	±0.9	±1.2	±1.1	±0.7	±0.5	±1.0	±0.7	±0.8

**Table 4:** Principal component loadings and total cumulative variance are explained by the first five principal components.

	PC 1	PC 2	PC 3	PC 4	PC 5
Whole milk δ^15^N	0.33	0.32	0.17	−0.29	0.59
Milk casein δ^15^N	0.24	0.30	−0.55	0.64	0.07
Milk whey δ^15^N	0.36	0.37	−0.08	−0.25	0.25
Whole milk δ^13^C	0.40	−0.32	0.14	0.20	0.05
Milk fat δ^13^C	0.39	−0.33	−0.05	0.15	0.03
Milk casein δ^13^C	0.36	−0.24	0.16	−0.28	−0.26
Milk whey δ^13^C	0.41	−0.29	0.05	0.12	−0.05
Whole milk d^2^H	0.11	0.45	0.72	0.41	−0.29
Whole milk δ^18^O	0.28	0.34	−0.31	−0.34	−0.66
Cumulative variability %	48.22	68.61	77.21	85.51	91.50

**Table 5:** The numbers of samples correctly assigned to the agroclimatic zones by the LDA analysis of stable isotope ratios. Numbers in parentheses provide the cross-validation results of the same using leave-one-out cross-validation method.

Agroclimatic Region of Authentic Samples and the Sample Number	Geographical Origins Correctly Defined by the LDA Model			The Accuracy of the LDA Model		
	Coconut Triangle	Dry Zone	Mid Country	Up Country	Before Cross-Validation	After Cross-Validation
Coconut triangle *n* = 36	30(27)	3(5)	3(4)	0	83%	75%
Dry zone *n* = 38	2(2)	35(35)	1(1)	0	92%	92%
Mid country *n* = 36	3(3)	0(1)	32(29)	1(3)	89%	81%
Up country *n* = 32	0	0	1(3)	31(29)	97%	91%

**Table 6:** Variation of mean values of trace elements in milk samples in each agroclimatic region. Data represent the mean ± standard deviation (μg L^−1^). Values in each row with different superscript letters differ for *p* < 0.001 (after [20]).

	Coconut Triangle	Dry Zone	Mid Country	Up Country
Li	5.21 ± 1.66 ^A^	1.00 ± 0.75 ^C^	3.55 ± 1.06 ^B^	0.53 ± 0.29 ^C^
Al	2251 ± 437 ^A^	479 ± 69.4 ^C^	2228 ± 247 ^A^	1194 ± 716 ^B^
Cr	477 ± 352 ^A^	19.8 ± 5.9 ^C^	305 ± 190 ^B^	31.3 ± 14.9 ^C^
Mn	159 ± 94 ^A^	31.8 ± 10.7 ^C^	117 ± 33.7 ^B^	24.3 ± 3.46 ^C^
Fe	3151 ± 1381 ^A^	486 ± 151 ^B^	2979 ± 1059 ^A^	723 ± 266 ^B^
Co	12.8 ± 11.4 ^A^	0.46 ± 0.24 ^B^	4.61 ± 2.34 ^B^	0.79 ± 0.47 ^B^
Ni	249 ± 224 ^A^	7.24 ± 1.8 ^B^	183 ± 166 ^A^	37.6 ± 28.5 ^B^
Cu	109 ± 43.5 ^A^	25.0 ± 9.29 ^B^	117 ± 37.2 ^A^	17.5 ± 14.6 ^B^
Zn	2925 ± 334 ^A^	1491 ± 351 ^B^	3089 ± 658 ^A^	1747 ± 342 ^B^
As	13.7 ± 3.21 ^A^	0.46 ± 0.3 ^B^	12.8 ± 2.92 ^A^	0.51 ± 0.6 ^B^
Se	28.0 ± 10.7 ^A^	12.0 ± 4.93 ^B^	20.8 ± 17.1 ^A^	9.46 ± 3.44 ^B^
Sr	669 ± 314 ^A^	427 ± 253 ^B^	488 ± 135 ^B^	185 ± 34.7 ^C^
Cd	2.47 ± 1.55 ^A^	1.25 ± 0.86 ^B^	1.23 ± 0.79 ^B^	0.58 ± 0.2 ^B^
Ba	426 ± 112 ^A^	260 ± 68.8 ^B^	417 ± 98.4 ^A^	194 ± 136 ^B^
Pb	20.4 ± 5.1 ^A^	5.01 ± 2.9 ^B^	16.3 ± 5.15 ^A^	9.02 ± 6.81 ^B^
Bi	1.04 ± 0.71 ^BC^	2.73 ± 3.47 ^BA^	0.34 ± 0.14 ^C^	3.38 ± 4.89 ^A^

## Data Availability

The data that support the findings of this study are available from the corresponding author upon reasonable request.

## Supplementary Materials

The following supporting information can be downloaded at: https://www.mdpi.com/article/10.3390/foods11030275/s1, Table S1: The whole dataset of authentic milk sample analysis including the description of the location, Stable isotopes ratios of C, N, H, and O, and elemental fingerprints of milk samples determined by IRMS and ICP-MS respectively.
